# Construction and validation of a prognostic model for hepatocellular carcinoma: Inflammatory ferroptosis and mitochondrial metabolism indicate a poor prognosis

**DOI:** 10.3389/fonc.2022.972434

**Published:** 2023-01-05

**Authors:** Fang Han, Dan Cao, Xin Zhu, Lianqiang Shen, Jia Wu, Yizhen Chen, Youyao Xu, Linwei Xu, Xiangdong Cheng, Yuhua Zhang

**Affiliations:** ^1^ Hepatobiliary and Pancreatic Surgery Department, The Cancer Hospital of the University of Chinese Academy of Sciences (Zhejiang Cancer Hospital), Institute of Basic Medicine and Cancer(IBMC), Chinese Academy of Sciences, Hangzhou, Zhejiang, China; ^2^ College of Food and Pharmacy, Zhejiang Ocean University, Zhoushan, Zhejiang, China; ^3^ Hepatobiliary and Pancreatic Surgery Department, Shaoxing Peoples’s Hospital, Shaoxing, Zhejiang, China; ^4^ Department of General Surgery, The First People’s Hospital of Linping District, Hangzhou, Hangzhou, Zhejiang, China; ^5^ Clincal Dept. Zhejiang Chinese Medical University, Hangzhou, Zhejiang, China

**Keywords:** hepatocellular carcinoma, prognostic model, inflammation, ferroptosis, mitochondrial metabolism, bioinformatics, survival analysis

## Abstract

**Background:**

An increasing number of innovations have been discovered for treating hepatocellular carcinoma (HCC or commonly called HCC) therapy, Ferroptosis and mitochondrial metabolism are essential mechanisms of cell death. These pathways may act as functional molecular biomarkers that could have important clinical significance for determining individual differences and the prognosis of HCC. The aim of this study was to construct a stable and reliable comprehensive model of genetic features and clinical factors associated with HCC prognosis.

**Methods:**

In this study, we used RNA-sequencing (fragments per kilobase of exon model per million reads mapped value) data from the Cancer Genome Atlas (TCGA) database to establish a prognostic model. We enrolled 104 patients for further validation. Gene Ontology (GO) and Kyoto Encyclopedia of Genes and Genomes enrichment analyses (KEGG) analysis were used for the functional study of differentially expressed genes. Pan-cancer analysis was performed to evaluate the function of the Differentially Expressed Genes (DEGs). Thirteen genes were identified by univariate and least absolute contraction and selection operation (LASSO) Cox regression analysis. The prognostic model was visualized using a nomogram.

**Results:**

We found that eight genes, namely EZH2, GRPEL2, PIGU, PPM1G, SF3B4, TUBG1, TXNRD1 and NDRG1, were hub genes for HCC and differentially expressed in most types of cancer. EZH2, GRPEL2 and NDRG1 may indicate a poor prognosis of HCC as verified by tissue samples. Furthermore, a gene set variation analysis algorithm was created to analyze the relationship between these eight genes and oxidative phosphorylation, mitophagy, and FeS-containing proteins, and it showed that ferroptosis might affect inflammatory-related pathways in HCC.

**Conclusion:**

EZH2, GRPEL2, NDRG1, and the clinical factor of tumor size, were included in a nomogram for visualizing a prognostic model of HCC. This nomogram based on a functional study and verification by clinical samples, shows a reliable performance of patients with HCC.

## Introduction

Liver hepatocellular carcinoma (HCC) is the third leading cause of cancer-related death worldwide (1). Most patients with HCC have a history of chronic hepatitis B virus (HBV) infection. Although HBV has been effectively controlled in China, morbidity and mortality are currently rising (2). Nevertheless, HCC is the sixth most common cancer worldwide (3). Several innovative methods have been developed for the treatment of HCC. However, the effectiveness of these treatments markedly varies between individuals. The main reason for this variation is a lack of therapeutic evaluation of efficacy. Therefore, more effective biomarkers and/or prognostic models are requied. Effective biomarkers should be able to predict the prognosis of HCC and participate in specific functions of tumor biology. In particular, biomarkers described for specific anti-tumor phenotypes may be the most meaningful (4, 5).

Intracellular iron is a crucial target for the induction of cell death because it mediates ferroptosis and mitochondrial metabolism (6). Ferroptosis is a non-apoptotic regulated form of cell death that has gradually attracted attention in cancer pathogenesis research (7). Ferroptosis induces abnormal mitochondrial metabolism and reactive oxygen species (ROS) accumulation, and it may promote anti-tumor mechanisms in the microenvironment (8, 9). These finding suggests that ferroptosis is an important mechanism contributing to the development of the tumor microenvironment. Some researchers have suggested that ferroptosis or metabolic-related gene signatures could provide relevant information on the prognosis of HCC (10, 11). The unique tumor microenvironment in HCC includes considerable immune infiltration because of chronic inflammation (12). Therefore, a prognostic model for HCC in the context of inflammation in mainland China is required.

In this study, a bioinformatics analysis method was used to analyze a data set from the Cancer Genome Atlas (TCGA) database to determine the differentially expressed genes (DEGs) in HCC compared with healthy individuals. A prognostic model based on the hub genes was constructed using least absolute contraction and selection operation (LASSO) regression. To determine the validity and stability of this model, we used clinical tissue samples to verify the hub genes. To verify the function of these critical genes in tumors, we performed a series of functional analyses. Using these results, we hoped to identify the most stable and reliable gene signatures and clinical factors that contribute to the progression of HCC.

## Materials and methods

### Data sources

Our analysis results are based on the omics data set generated by the TCGA (http://cancergenome.nih.gov/). A total of 33 types of cancer were enrolled from TCGA. All data sets include Count data, Transcripts Per Kilobase Million (TPM) data, RNA-seq, Copy number variation (CNV), Mutation and Clinical Information, were downloaded from UCSC XENA (https://xenabrowser.net/). We enrolled a total of 50 normal liver tissues and 374 HCC in the TCGA database. We used the limma package for R to analyze the differential expression between HCC and normal liver tissues.

### Clustering differentially expressed genes for HCC

To identify the Fold Changes in gene expression in each cancer data set, we used the Deseq2 package for R software to identify the DEGs using TCGA count data. We consider adjusting P value < 0.05 and Log|FC| > 1 as DEGs. Meanwhile, to determine the DEGs related to the prognosis of HCC, we used univariate COX regression, which is analyzed in R using survival package to screen the genes that affect the prognosis of HCC. HCC-specific genes are considered meaningful to both differential expression and prognostic analysis.

### GO and KEGG analysis

Gene Ontology (GO) and Kyoto Encyclopedia of Genes and Genomes Enrichment Analyses were functional enrichment. Go analysis is a bioinformatics tool including three functional analyses, which are cellular components (CC), molecular function (MF), and biological pathways (BP) (13). KEGG stores a multigene signature pathway to which hub genes belong (14). We conducted a functional enrichment analysis to study the underlying mechanism of DEGs-based clustering at different levels. We first converted the gene names of the differential genes into ENTREZ IDs, and further, enriched using the enrichment function in the cluterprofiler. For the P value of the results of the analysis, the BH algorithm is used to correct it. Finally, we select the corrected P value of < 0.05 as the meaningful enrichment result

### LASSO Cox regression analysis and visualization

We first performed COX regression on differentially expressed genes to screen genes related to the prognosis of HCC. The LASSO regression analysis model eliminates the collinearity problem between prognostic-related genes (15). Finally, we obtained the characteristic genes that affect HCC. We used a nomogram, which constructed specific models related to HCC for clinical application to show the clinical model. Lasso returns to the Glmnet package using the R language. We used the Rms package to construct the nomogram graph.

### Patients and tissue samples collection

A total of 104 HCC patients were enrolled in The Cancer Hospital of the University of Chinese Academy of Sciences(Zhejiang Cancer Hospital) from 2011 to 2020. Each patient was collected one pair of normal and cancer tissue within 30 minutes after surgical resection. *Normal tissue* was defined as greater than 1cm beyond the tumor margin. A frozen section was identified by senior pathologists, who were more than 15 years of practitioners, to exclude tumor lesions under the microscope. HCC lesions, which senior pathologists diagnosed, were acquired about 1cm inside the tumor margin. All patients were pathologically confirmed as Hepatocellular carcinoma. The clinical stage of the tumor was determined according to the Cancer Staging Manual of the American Joint Committee on Cancer(version 8, 2017), while the Edmondson Steiner classification defined the tumor pathological differentiation stage. All 104 patients were enrolled, including 19 females and 85 males. There were 58 patients older than 60 years old and another 46, respectively. The follow-up ended in March 2021 or at death. HCV infections were excluded in our study.

### Immunohistochemistry stain

All 104 pairs of lesions were paraffin-fixed tissue. All slice tissue was deparaffinized, rehydrated, and then heated in citric acid buffer (0.01M) at 105°C for 10 min. After antigen recovery, the sliced tissue was blocked with 3% hydrogen peroxide solution and bovine serum albumin. Subsequently, the slides were incubated with rabbit antibodies, including anti-EZH2, anti-GRPEL2, anti-PIGU, anti-PPM1G, anti-SF3B4, anti-TCOF1, anti-TUBG1, anti-TXNRD1, anti-MYCN, anti-NDRG1, anti-SQSTM1, anti-UCK2, at 4°C overnight, then incubated with horseradish peroxidase (HRP)-conjugated secondary antibody at room temperature for 20 min. Finally, the slides were counterstained with hematoxylin, dehydrated in graded alcohol and xylene, cleared, and mounted. The antibody or catalogue and dilutions are listed in **Supplementary Table 1**.

### IHC quantification

All slices were measured by Pannoramic DESK II DW Digital Slide Scanner(Coherent Scientific Ltd.). After the tissue section was set on the scanner, the section gradually moved under the scanner lens. While moving, all tissue information would be scanned and recorded to form a folder containing all image details. The folder was opened by using CaseViewer2.2 software. It can be magnified at any multiple of 1x-400x for observation. Use the TMA plug-in in the Quant Center2.1 analysis software to set the diameter of the chip organization point and the number of rows and columns. The software will automatically generate the number. Use the PatternQuant module in the Quant Center2.1 analysis software to distinguish the brown area (including positive) from the blue part. The two parts of the Mask Area are used as the tissue area; add HistoQunant is merged in the next layer of the brown area. Finally, the area and grayscale of the positive part are calculated based on the optical density.

### Pan-cancer analysis for DEGs

To determine the role of the above 13 DEGs, we used the genes to perform pan-cancer analysis to observe the expression, mutation frequency, and copy number change frequency of these genes in pan-cancer. All TCGA pan-carcina data can be downloaded from UCSC XENA. In gene mutation analysis, we calculated the proportion of mutations in each gene in different cancer samples to compare which gene was more likely to mutate. In copy number analysis, the proportion of each gene that increases or decreases in copy number is calculated. We observed changes in gene copy number using the increase in copy number and the decrease in ratio. Finally, we used Deseq2 to calculate the differential expression of each gene. The differential expression of core genes in multiple cancers and the effect of gene expression on the prognosis of different tumors were observed.We used TCGA+GTEx and GSE101685 to validate the core genes. TCGA+GTEx contains 369 cancers and 160 normals, while GSE101685 includes 24 cancer samples and eight normal samples.

### Identification of hub genes and functional enrichment analysis

The Gene Set Variation Analysis(GSVA) algorithm is an algorithm that obtains phenotypic scores based on the amount of gene expression associated with different phenotypes. We first collected genes associated with each immune cell. Further, based on the expression of these genes, each immune cell score is obtained. First, we used the Gene Set Variation Analysis(GSVA) to calculate the impact of 24 types of immune cells on HCC. The relationship between core genes and immune cell scores was analyzed. Pearson correlation analysis was further used for the relationship between hub genes and immune cells in HCC. To investigate the relationship between risk score and immune checkpoints, we extracted the expression of 30 immune checkpoints, including the B7-CD28 family (CD274, CD276, CTLA4, HHLA2, ICOS, ICOSLG, PDCD1, PDCD1LG2, TMIGD2, VTCN1), TNF superfamily (BTLA, CD27, CD40LG, CD40, CD70, TNFRSF18, TNFRSF4, TNFRSF9, TNFSF14, TNFSF4, TNFSF9), and another immune checkpoint (HAVCR2, IDO1, LAG3, FGL1, ENTPD1, NT5E, SIGLEC15, VSIR, NCR3). Furthermore, we measured the coexpression relationship between DEGs and Ferroptosis. As all we know, cell death is affected by mitochondria dysfunction generally. Therefore, we downloaded the data from MITOCARTA 3.0 to evaluate mitochondria-related pathways. The GSEA algorithm was used to analyze the effect of DEGs on OXPHOS, Mitophagy and Fe.S containing proteins.

### Identification of hub genes and drug sensitivity and regulatory mechanisms

We assessed the drug sensitivity of each patient using pRRophetic (R package, University of Minnesota System), and further used correlation analysis to relate the relationship between prognostic models and tumor drug sensitivity. pOSTAR3 is a database used to predict post-transcriptional regulation of genes, and to understand the transcriptional regulatory relationships of three iron death-associated genes, we constructed a post-transcriptional regulatory network using POSTAR3. GSE106988 is a dataset of RNA-seq performed after knockdown of NDRG1. Using this database. We analyzed the potential functions of NDRG1 and used cytoscape software for the visualization of protein pathway construction.

### Statistical analysis

R (version 3.6.2, www.r-project.org) and relative packages for R were used for bioinformatics and statistical analysis. SPSS (Version 25.0) was used for statistical analysis. Visualization mainly used the R package ggplot2(version 3.3.3). The chi-square test and Fisher’s exact test were used to evaluate the statistical significance of the relationship between DEGs expression and clinicopathological parameters. The Student’s unpaired t-test is used to express customarily distributed DEGs; otherwise, the Mann-Whitney U test is used. The Wilcoxon signed-rank test was used to analyze the difference between HCC and adjacent non-HCC lesion distribution. Survival curves were assessed by the Kaplan-Meier method and Cox proportional hazards regression model, and a log-rank test analyzed the differences. Before performing the Kaplan-Meier analysis, the Best Separation method was used to cut gene expression into high and low groups. *P* < 0.05 was considered as statistical significance. After building the model, we evaluated our model by C-index and decision curve.

## Results

### Identification of DEGs from the TCGA

**Figure 1:**
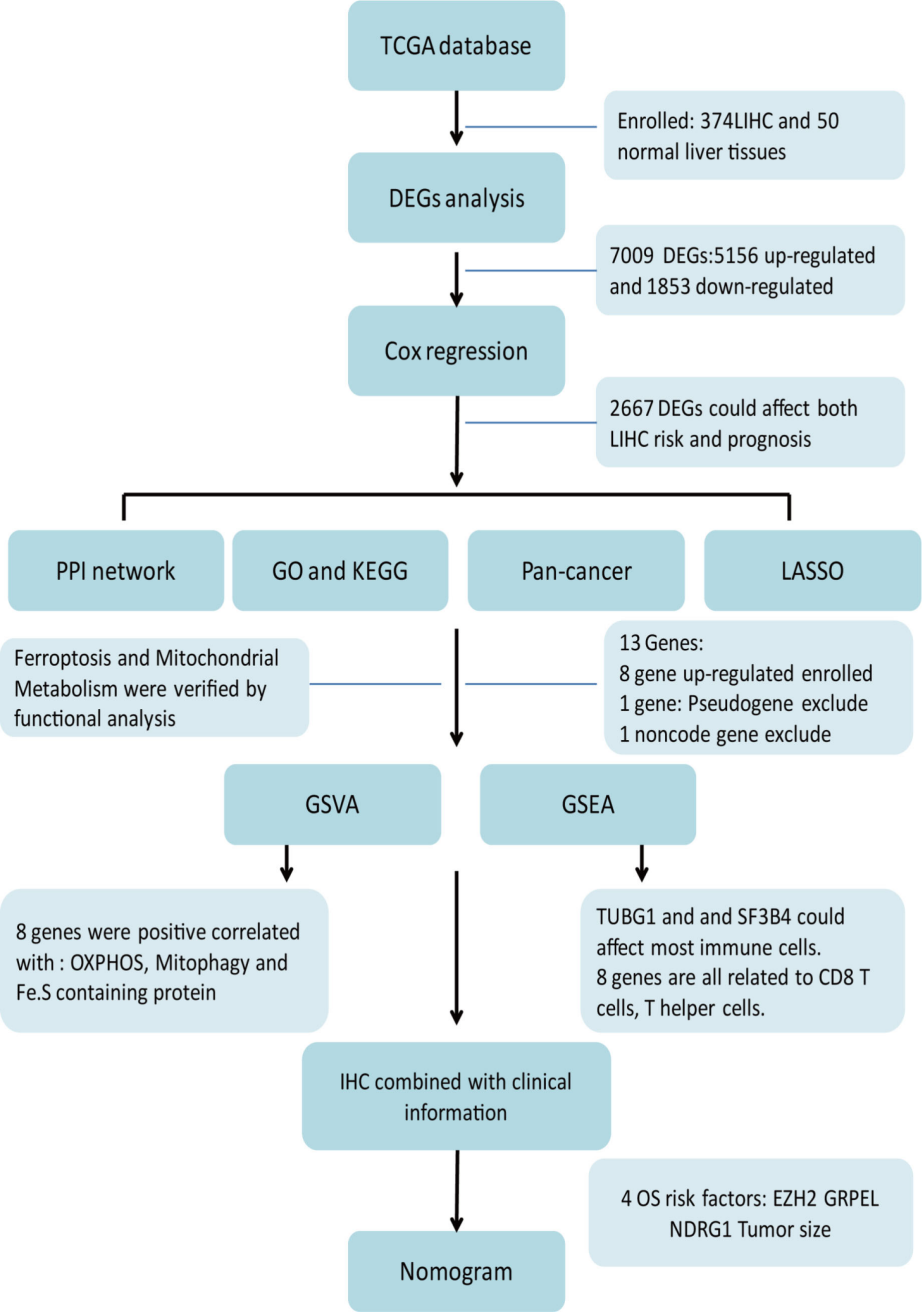
Workflow-process diagram of the present study.

**Figure 2:**
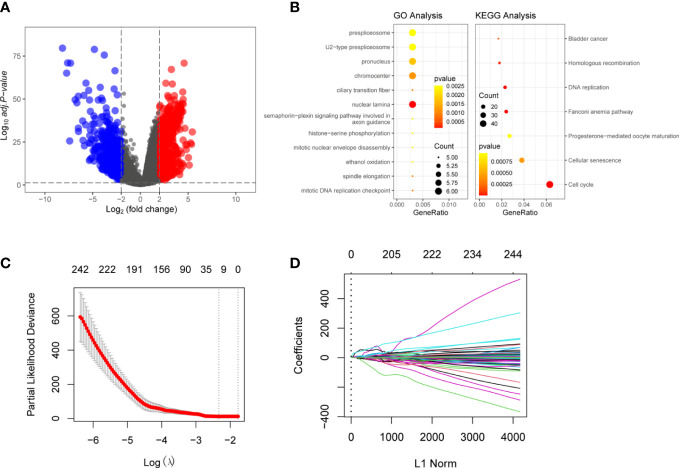
Identification of The Differentially Expressed Genes (DEGs) in HCC and normal liver tissues: **(A)** Volcano plot of the DEGs in TCGA dataset. Log|FC|>2 was considered as DEGs In the figure, red represents high-expression genes, and blue represents low-expression genes **(B)** Functional analyzing by GO and KEGG. The yellower the color indicates that the result is more meaningful, and the larger the circle, the more genes are enriched within the result. **(C)** LASSO Cox regression was performed to identify the DEGs related to the prognosis of HCC (λ = 0.08869376). **(D)** Visulization of LASSO Cox regression. These 13 genes that were DSTNP2, EZH2, GRPEL2, 227 MYCN, NDRG1, PIGU, PPM1G, SF3B4, SQSTM1, TCOF1, TUBG1, TXNRD1, and UCK2 might predict prognosis of HCC.

The entire analysis flow of the manuscript results is shown in **Figure 1**. DESeq2 was performed for DEG analysis using RNA-sequencing fragments per kilobase of the exon model per million mapped fragment data from the HCC TCGA database. We found a total of 7009 DEGs, of which 5156 genes were up-regulated and 1853 genes were downregulated (**Figure 2A**). Furthermore, to determine the genes that affect the risk and prognosis of HCC, we performed survival analysis by Cox regression for all 7009 genes. We found that 2667 genes might affect the risk of HCC and its prognosis.

### Function analysis of DEGs by Gene Ontology and Kyoto Encyclopedia of Genes and Genomes

To better understand the function of the 2667 DEGs, we performed Gene Ontology (GO) and Kyoto Encyclopedia of Genes and Genomes (KEGG) functional enrichment analysis. The GO enrichment analyses. The GO enrichment analysis showed 387 items were related to the DEGs, such as histone−serine phosphorylation and mitotic DNA replication checkpoint. KEGG analysis showed that the DEGs were mainly involved in the nuclear lamina prespliceosome mitotic DNA replication checkpoint. Other pathways included cellular senescence, DNA replication, and oxidation (**Figure 2B**). **Supplementary Tables 2**, **3** show more details of GO and KEGG analyses.

### Risk scoring model for the application of the LASSO algorithm for HCC

We used LASSO regression to eliminate 2,667 covariates of DEGs that affect the risk of HCC and its prognosis (16). We screened the LASSO regression model using λ = 0.08869376 (**Figure 2C**). The LASSO regression visualization results are shown in **Figure 2D**. Finally, 13 hub genes that might predict the prognosis of HCC were obtained. These 13 genes that were DSTNP2, EZH2, GRPEL2, MYCN, NDRG1, PIGU, PPM1G, SF3B4, SQSTM1, TCOF1, TUBG1, TXNRD1, and UCK2. The details of these hub genes are shown in **Tables 1**, **2**. We excluded the pseudogene DSTNP2 because we were more interested in protein expression genes. In addition, we chose eight upregulated genes in HCC and compared with healthy tissues as hub genes. These genes were TXNRD1, TUBG1, SF3B4, PPM1G, PIGU, NDRG1, GRPEL2 and EZH2.

**Table 1:** Details of the Hub genes.

Gene Symbol	Gene name	Ensembl ID	Transcript Biotype	Chromosome	Start Position	End Position	Band
DSTNP2	Destrin Pseudogene 2	ENSG00000248593	processed_transcript	12	6884682	6885786	p13.31
EZH2	Enhancer of zeste 2 polycomb repressive complex 2 subunit	ENSG00000106462	nonsense_mediated_decay	7	148807257	148884321	q36.1
GRPEL2	GrpE Like 2, Mitochondrial	ENSG00000164284	protein_coding	5	149345430	149354583	q32
MYCN	MYCN Proto-Oncogene	ENSG00000134323	protein_coding	2	15940550	15947007	p24.3
NDRG1	N-Myc Downstream Regulated 1	ENSG00000104419	protein_coding	8	133237175	133302022	q24.22
PIGU	Phosphatidylinositol Glycan Anchor Biosynthesis Class U	ENSG00000101464	protein_coding	20	34560542	34698790	q11.22
PPM1G	Protein Phosphatase, Mg2+/Mn2+ Dependent 1G	ENSG00000115241	retained_intron	2	27381195	27409591	p23.3
SF3B4	Splicing Factor 3b Subunit 4	ENSG00000143368	protein_coding	1	149923317	149927803	q21.2
SQSTM1	Sequestosome 1	ENSG00000161011	processed_transcript	5	179806398	179838078	q35.3
TCOF1	Treacle Ribosome Biogenesis Factor 1	ENSG00000070814	protein_coding	5	150357629	150400308	q32
TUBG1	Tubulin Gamma 1	ENSG00000131462	retained_intron	17	42609641	42615238	q21.2
TXNRD1	Thioredoxin Reductase 1	ENSG00000198431	protein_coding	12	104215779	104350307	q23.3
UCK2	Uridine-Cytidine Kinase 2	ENSG00000143179	protein_coding	1	165827614	165911618	q24.1

Obtained from National Center of Biotechnology Information (NCBI) database.

**Table 2:** Hazard ratio of 14 hub genes.

gene	HR	95%CI	p	p.val
EZH2	1.144	1.096 - 1.195	9.71E-10	< 0.001
UCK2	1.034	1.025 - 1.044	1.88E-12	< 0.001
PIGU	1.078	1.048 - 1.108	1.27E-07	< 0.001
SF3B4	1.014	1.01 - 1.019	2.25E-10	< 0.001
TCOF1	1.037	1.026 - 1.049	1.01E-10	< 0.001
DSTNP2	4.334	2.892 - 6.495	1.21E-12	< 0.001
TUBG1	1.02	1.013 - 1.028	1.41E-07	< 0.001
GRPEL2	1.261	1.179 - 1.347	9.94E-12	< 0.001
PPM1G	1.015	1.01 - 1.019	1.32E-10	< 0.001
MYCN	1.177	1.109 - 1.248	5.87E-08	< 0.001
TXNRD1	1.002	1.001 - 1.003	3.52E-12	< 0.001
SQSTM1	1.103	1.001-1.003	1.53E-10	< 0.001
NDRG1	1.001	1 - 1.001	5.79E-07	< 0.001

### Pan-cancer analysis of DEGs

**Figure 3:**
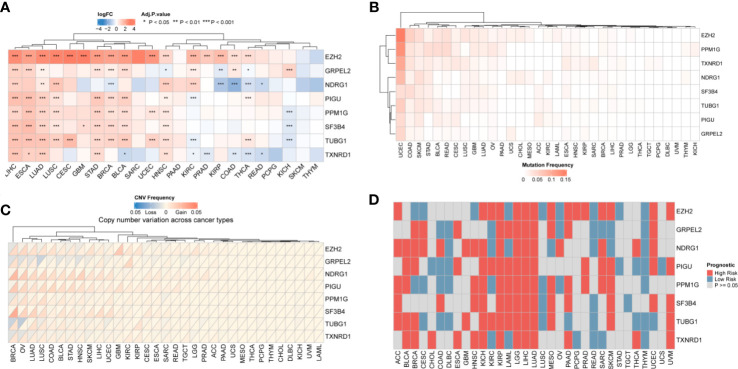
Pan-cancer results of hub genes. **(A)** Differential Expression of genes: eight genes were differentially expressed in most cancer types. The darker the color, the greater the fold change **(B)** Mutation frequency: the hub gene mutation was relative stable in all types of cancer except UCEC. **(C)** Copy number variation of hub genes. HCC had minor changes in the copy number of the 8 hub genes **(D)** Prognosis of hub genes. The red square represents a risk factor in the cancer, and the blue square represents a protective factor. Gray indicates that the prognosis of the tumor does not affect the prognosis.

The eight hub genes upregulated in HCC mentioned above were differentially expressed as shown by pan-cancer analysis (**Figure 3A**) These eight genes were differentially expressed in digestive tract tumors, such as stomach adenocarcinoma and esophageal carcinoma. GRPEL2 and EZH2 were highly expressed in most cancer types, such as HCC, esophageal carcinoma, and stomach adenocarcinoma. NDRG1 was also highly expressed in HCC, lung squamous cell carcinoma, and head and neck squamous carcinoma. Uterine corpus endometrial carcinoma was associated with genetic mutations in the analyzed samples, and there were only a few mutations in the remaining types of cancer (**Figure 3B**). In addition, we performed a copy number alteration analysis and showed that most cancer types, inculding HCC, had minor changes in the copy number (**Figure 3C**). These findings indicated that these eight hub genes were characteristic genes of HCC, and their expression may not be affected by other genomic changes. We then examined the effect of these eight genes on the prognosis of all cancers. We found that these eight genes might affect the prognosis of LGG, HCC, and LUAD. Therefore, a high expression of these genes is a risk factor for these cancers (**Figure 3D**).

### Functional analysis of genes

**Figure 4:**
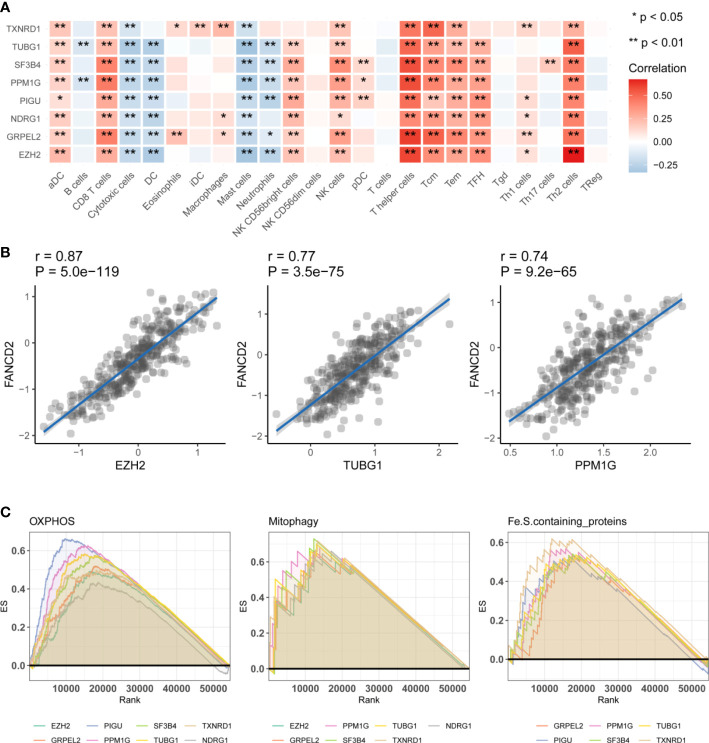
Functional analysis of the hub genes. **(A)** Immunoinfiltration analysis. Gene Set Variation Analysis were used to calculate the effect of 24 types of immune cells on HCC. Significance is presented by using *p < 0.05, **p < 0.01. The darker the red color, the higher the correlation coefficient. **(B)** The correlation analysis between hub genes and ferroptosis related genes FANCD2. EZH2, TUBG1, PPM1G were significantly positive effective with FANCD2. **(C)** The correlation analysis of three hub genes, EZH2, GRPEL2, NDRG1, and FANCD2. Gene Set Enrichment Analysis, (GSEA) the hub genes were enriched in OXPHOS, Mitophagy, Fe.S.containing proteins. It has been partially confirmed that ferroptosis and mitochondrial metabolism were related to prognosis in HCC.

We conducted a functional analysis of the eight genes described above. First, we used the Gene Set Variation Analysis (GSVA) to calculate the effect of 24 types of immune cells on HCC. This was performed by comparing the correlations between the eight hub genes and immune cells. We found that TUBG1 and SF3B4 could affected most immune cells, and eight of the hub genes were positively related to CD8 T cells and T helper cells. These findings indicated that these eight genes might affect the prognosis of HCC by affecting T helper cells (**Figure 4A**). Eight of the immune checkpoint genes were related to TNFRSF9, TNFSF9, HAVCR2, and ENTPD1 (**Supplementary Table 8** and **Supplementary Figure 5**). We also analyzed the correlations between the eight hub genes and ferroptosis-related genes. The results showed that the eight genes are positively correlated with most of the ferroptosis-related genes (**Supplementary Figure 1**, **Supplementary Table 5**). There was a strong positive correlation between FA complementation group D2(FANCD2), and three characteristic genes, namely EZH2, TUBG1, and PPM1G (**Figure 4B**). These results indicated that these eight hub genes may affect ferroptosis in HCC. We used a gene set enrichment analysis (GSEA) algorithm to analyze the relationship between the eight hub genes and mitochondria-related pathways. We found that oxidative phosphorylation (OXPHOS), apoptosis, mitophagy, and Fe.S-containing proteins were positively related to these genes according to the enrichment score (**Figure 4C**). The enrichment scores were positively related to OXPHOS, mitophagy, and Fe.S-containing proteins. On the basis of these results, we hypothesize that ferroptosis affects the prognosis of HCC *via* mitochondria-related mechanisms.

### Validation of gene expression differences and the clinical significance of the prognosis

**Figure 5:**
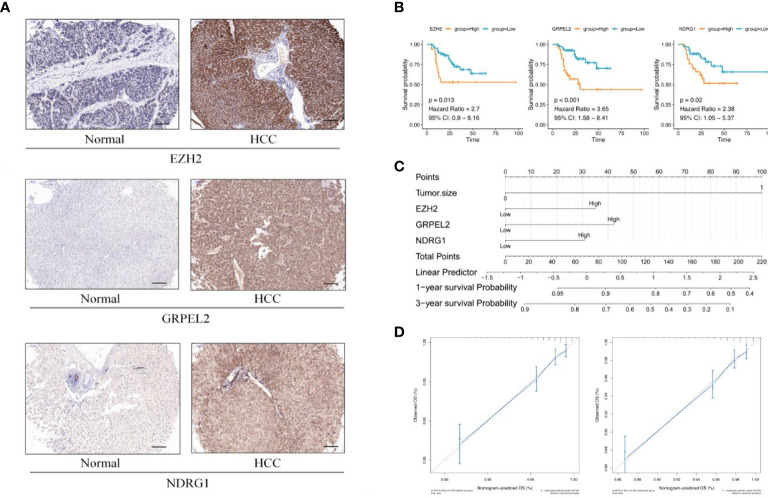
Establishment of prognostic model **(A)** Differential expression between HCC and adjacent non-HCC lesions. IHC image for EZH2, GRPEL2, NDRG1, paired with non-LIHC tissues and LIHC tissues. **(B)** KM curves of EZH2, GRPEL2, NDRG1. Higher expression of hub genes meant poor prognosis of HCC. **(C)** Nomogram was for visulizing the prognostic model. Three factors, EZH2, GRPEL2, NDRG1, and tumor size, were enrolled in the prognostic model to predict the survival rate of 1,3 years survival in HCC **(D)** The nomogram calibration curves of 1-, 3-year survival probabilities. The tool bar =100μm.

**Figure 6:**
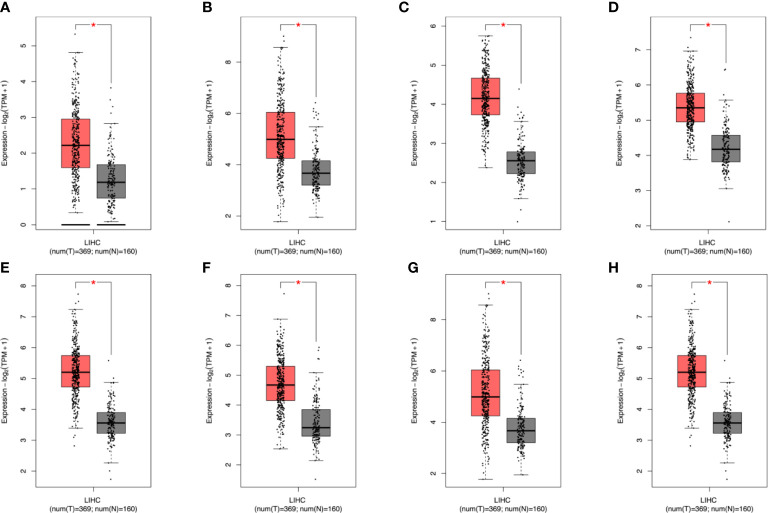
TCGA+GTEx and GSE101685 data to further analyze the differential expression of genes. After analysis, it was found that the eight genes verified above were all differentially expressed. In the figure, red represents cancer patients and gray represents normal samples **(A–H)** EZH2,GRPEL2, PIGU, PPM1G, SF3B4, TXNRD1, NDRG1, TUBG1. * stands for *P* value < 0.05.

We measured the expression of the 13 identified hub genes (EZH2, GRPEL2, MYCN, NDRG1, PIGU, PPM1G, DSTNP2, SF3B4, SQSTM1, TCOF1, TUBG1, TXNRD1, and UCK2) by immunohistochemistry (IHC) in tissue samples of 104 patients. We found that eight genes, EZH2, GRPEL2, PIGU, PPM1G, SF3B4, TUBG1, TXNRD1, and NDRG1, were expressed differently between HCC and adjacent non-HCC (**Supplementary Table 5**, **Supplementary Figures 2, 3** and **Figure 5A**). These results were consistent with bioinformatics analysis. Moreover, the expression levels of these genes were significantly different between HCC and adjacent non-HCC lesions (**Supplementary Table 5**). In conclusion, these eight genes could be used as potential risk factor indicators to determine clinical indications for prognosis. To further ensure the accuracy of core genes, we used TCGA+GTEx and GSE101685 data to further analyze the differential expression of these genes. We found that the eight hub genes identified above were all differentially expressed (**Figure 6** and **Supplementary Table 7**).

### HCC prognosis-related model construction

In addition to analyzing expression of the eight hub genes, we collected basic information on all 104 patients, such as sex, age, histological differentiation, vascular cancer embolus, cirrhosis, tumor size (cm), alpha-fetoprotein concentrations (ng/mL), hepatitis B surface antigen concentrations, alanine aminotransferase, underlying diseases, and total bilirubin concentrations. We excluded hepatitis C viruse infection patients in our study. These clinical factors are generally considered indicators of the prognosis of HCC. In our dataset, the one-factor survival analysis showed that a larger tumor size and positive vascular cancer embolus indicated a poor prognosis. Therefore, we incorporated these factors into the Cox regression model in the multivariate analysis. The high expression of EZH2, GRPEL2, TCOF1 and NDRG1 genes suggested a poor prognosis, as shown in the multivariate regression analysis (**Table 3**, **Figure 5B**). Furthermore, a nomogram was used for visualization to better use as a clinical prognostic model. The nomogram showed that a larger tumor size and higher EZH2, GRPEL2, and NDRG1expression predicted the higher likelihood of a worse prognosis (**Figure 5C**). The nomogram was able to predict the probability of 1- and 3-year survival (**Figure 5D**). These findings indicated a close relation to survival, which suggested that our nomogram may be a reliable prognostic model for HCC. Furthermore, we computed the C-index to evaluate our model. The C-index of this model was 0.811(0.797-0.825). A decision curve showed that this model had reliable clinical practicability (**Supplementary Figure 4**).

**Table 3:** Survival analysis combined with clinical parameter by Immunosotochemistry.

Variability	n	One-factor cox regression model		Multi-factor cox regression model	
		HR(CI 95%)	*P*	HR(CI 95%)	*P*
**Gender**					
Female	19	2.218(0.968, 5.086)	0.060		
Male	85				
**Age**					
>60	58	0.917(0.428,1.965)	0.823		
<=60	46				
**Histological differentiation**					
Poorly	29	0.606(0.229, 1.603)	0.313		
Well	75				
**Microvascular cancer embolus**					
Positive	65	0.433(0.198,0.943)	0.035	1.661(0.739,3.731)	0.219
Negative	39				
**Cirrhosis**					
Positive	64	0.565(0.265,1.204)	0.139		
Negative	40				
**Tumor size (cm)**					
≤5	42	6.167(2.487,15.293)	8.60E-05	5.358(2.126,13.506)	3.73E-04
> 5	62				
**AFP (ng/mL)**					
> 40	41	0.882(0.403,1.931)	0.754		
≤40	63				
**ALT (U/L)**					
> 40	39	1.649(0.772,3.520)	0.196		
≤40	65				
**Total bilirubin**					
> 17.1	45	1.680(0.787,3.585)	0.180		
≤17.1	59				
**Albumin**					
≤40	57	1.258(0.584,2.711)	0.558		
> 40	40				
**EZH2**					
high	87	0.368(0.161,.843)	0.018	0.272(0.113,0.659)	0.004
low	17				
**GRPEL2**					
high	70	0.272(0.126,0.587)	0.001	0.321(0.148,0.697)	0.004
low	34				
**PIGU**					
high	74	0.614(0.271,1.388)	0.241	0.663(0.286,1.537)	0.338
low	30				
**PPMIG**					
high	74	0.514(0.226,1.171)	0.113	0.676(0.285,1.606)	0.375
low	30				
**DSTP2**					
high	47	1.252(0.588,2.669)	0.560	0.743(0.336,1.647)	0.465
low	57				
**SF3B4**					
high	70	0.885(0.383,2.041)	0.774	0.886(0.377,2.081)	0.780
low	34				
**TCOF1**					
high	52	0.683(0.319,1.465)	0.328	0.503(0.230,1.104)	0.087
low	52				
**TUBG1**					
high	41	1.641(0.770,3.499)	0.199	1.102(0.484,2.511)	0.817
low	63				
**txnRD1**					
high	42	1.091(.509,2.340)	0.823	0.714(0.311,1.641)	0.428
low	62				
**Mycn**					
high	49	0.577(0.266,1.254)	0.165	0.590(0.268,1.300)	0.191
low	55				
**NDRG1**					
high	70	0.420(0.197,0.894)	0.024	0.408(0.190,0.878)	0.022
low	34				
**SQSTM1**					
high	49	1.031(0.483,2.200)	0.938	1.078(0.501,2.321)	0.848
low	55				
**UCK2**					
high	49	0.924(0.433,1.971)	0.838	0.456(0.194,1.071)	0.072
low	55				

Tumor size and Microvascular cancer embolus was considered as the clinical parameters which effect prognosis. HR, hazard ratio; CI, confidence interval. *P* < 0.05 considered as statistical significance. Survival analysis combined with clinical parameter by Immunosotochemistry. We enrolled all 104 pairs of HCC and adjacent non-HCC lesions. All patients were HBsAg positive. There are ten clinical factors were enrolled, which are Gender, Age, Histological differentiation, Microvascular cancer embolus, Cirrhosis, Tumor size (cm), Alpha-fetoprotein (AFP) (ng/mL), Alanine aminotransferase (ALT), Total bilirubin, Albumin. EZH2, GRPEL2, NDRG1, Tumor size were independent prognostic risk factors. (P < 0.05) Albumin test results were missing in 2 of 104 patients.HCV infection was excluded in the cohort.

### HCC prognosis-related model functional analysis

**Figure 7:**
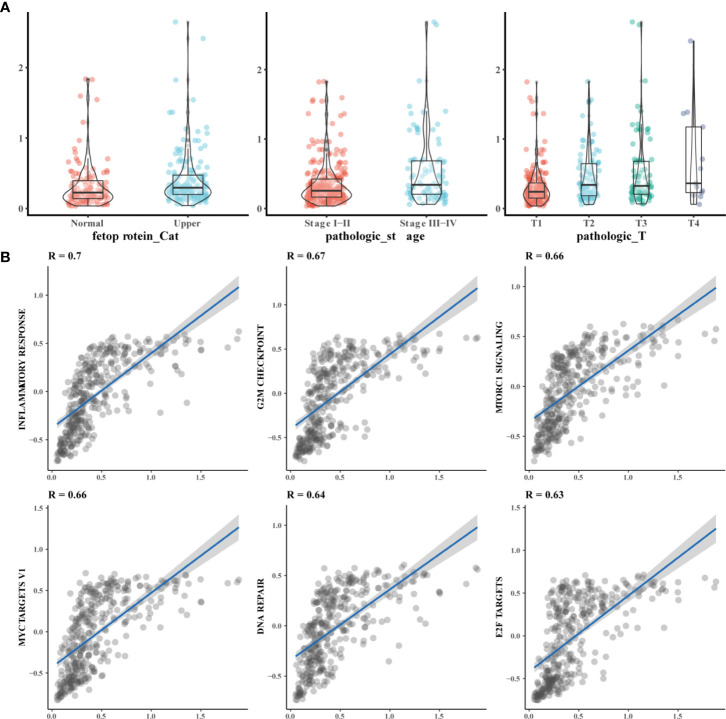
Functional of prognosis related model function. **(A)** The model score is correlated with the severe of clinical parameters Alpha-fetoprotein abnormalities, the higher the pathologic stage and the higher the T stage, the higher the prognostic model score; **(B)** The Model was mainly related to the inflammatory response and other pathways. GSVA scores were performed on tumor-related pathways, and the correlation between model scores and tumor-related pathways was observed through correlation analysis of pathway scores and model scores.

To further understand the function of the HCC prognostic model, we analyzed the relationship between model scores and clinical parameters. We found that the prognostic model score was mainly related to alpha-fetoprotein, T stage. Among them, the model score is higher when the disease is more severe (**Figure 7A**). In addition, we analyzed the relationship between 3 genes and HBV infection. EZH2 was related to HBV infection, and the expression of EZH2 expression was high in patients with HBV infection (**Supplementary Table 9**). In addition, GSVA scores were calculated for tumor-related pathways, and the correlations between model scores and tumor-related pathways were determined. We found that the HCC model was mainly related to the inflammatory response and other pathways (**Figure 7B**).

### Prognostic models and drug resistance

**Figure 8:**
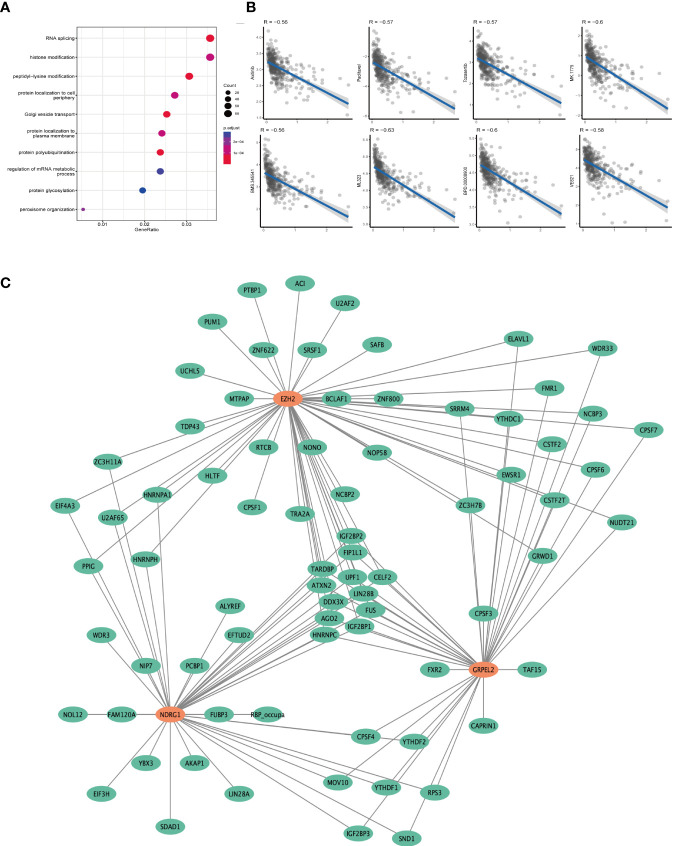
Prognostic model related related functional analysis. **(A)** Functional enrichment analysis results of NDRG1 knockout dataset. The bluer the color in the figure, the more meaningful the result. **(B)** The relationship between prognostic model and drug sensitivity. Each point in the graph represents the model score of a sample as well as the drug sensitivity score. **(C)** Post-transcriptional regulatory network of genes associated with prognostic models. The orange circle represents the gene in the model, and the green circle represents the poor post-transcriptional regulatory protein.

Our analysis using GSE106988 showed that NDRG1 was mainly associated with functions such as RNA shearing(**Figure 8A**), but the other two genes need to be verified in subsequent experiments. After analyzing the targets of anti-tumor drug sensitivity action in patients with HCC, we found that the prognostic model of ferroptosis was associated with multiple chemotherapy efficacy, with the strongest correlation with axitinib (**Figure 8B**). We also analyzed the function of the three core genes for ferroptosis and the regulatory relationships. Finally, to understand the post-transcriptional regulatory relationships of these three genes, analysis using POSTAR3 analysis showed that these three genes were affected by multiple post-transcriptional regulatory genes, among which IGF2BP2, FIPILI, TARDBP, ELF2, ATXN2, DDx3X, AGO2, IGF2BP1, and HNRNPC regulated these three genes simultaneously (**Figure 8C**).

## Discussion

HCC is a common and highly malignant tumor. Patients with HCC have a poor prognosis (17). At present, surgery is the most effective treatment method for HCC (18). However, at diagnosis, most patients with HCC show intra- and extra-hepatic metastases, vascular tumor thrombi and inadequate future liver volume, which indicate that the opportunity for surgery has already passed. With the development of advanced therapies, various emerging treatments have achieved positive results (19, 20). However, because of the complexity of the genetic background of HCC, the benefits of these treatments remain unclear (20, 21). Therefore, in-depth studies of the pathogenesis of HCC, including machine learning, will have clinical significance for predicting the interaction of hub genes and investgating prognostic-related molecular markers for individuals. In this study, we constructed a prognostic model combined with clinical information, functional analysis, and pan-cancer analyses to verify our bioinformatic results (22).

In this study, we used the TCGA data sets to predict cancer the risk of HCC and its prognosis. We found that the DEGs were highly related to DNA replication, oxidation, and inflammatory response. According to previous studies, these mechanisms are consistent with the activity of tumor cell growth and mitochondrial metabolism (23). Therefore, we speculate that abnormal mitochondrial function is related to the occurrence and development of HCC. The LASSO regression analysis was performed to generate a prognostic signature. We found 13 hub genes that were potentially related to the prognosis of HCC. Eight upregulated genes (EZH2, GRPEL2, PIGU, PPM1G, SF3B4, TUBG1, TXNRD1, and NDRG1) were not only found to be tumorigenesis risk factors, but also factors of a poor prognosis of HCC. In addition, we analyzed 104 clinical samples to validate our predictions. These eight genes were differentially expressed between HCC and adjacent non-HCC lesions, which suggested that these genes could be used as biomarkers for risk factors. The multivariate regression analysis showed that EZH2, NDRG1 and GRPEL2, as well as the tumor size were independent factors for the prognosis of HCC. According to recent surveys, TOCF1 (11) and UCK2 (24) may also indicate the prognosis in the patients with HCC. Unfortunately, our study did not show any statistical significance for the differentiated expression of TOCF1 or UCK2. Therefore, a larger sample size and multicenter study would be useful to further validate the relationship between the expression of these two genes and the prognosis of HCC.

We also constructed a prognostic nomogram model, which included EZH2, GRPEL2, NDRG1, and the tumor size. EZH2 is a transcriptional suppressor gene (25). EZH2 combines with EED, SUZ12, and RBBP4 to generate a PRC2 subunit, which is overexpressed in multiple types of cancer (26). EZH2 has a bidirectional tumorigenic effect involving mTOR signaling, as shown in EZH2-deficient leukemia cells (27). In addition, detecting EZ gene expression by tissue puncture biopsy can be used as a molecular marker for the prognosis of patients with HCC (28). There have been several recent clinical trials of EZH2 inhibitors in various cancers being studied (29–32). Our study showed that EZH2 was involved in autophagy, which is consistent with previous studies. EZH2 may also have specific effects on mitophagy, ferroptosis, and OXPHOS. Determining the specific mechanism of EZ2H should be considered in future research. NDRG1 plays a crucial role in angiogenesis, proliferation, invasion, and prognosis of HCC (33, 34). NDRG1 may also be a molecular marker for metastasis and prognosis in HCC. NDRG1 may predict the recurrence of HCC in liver transplant patients after surgery (35). This gene plays an anti-tumor role by involving the Wnt signaling pathway and other carcinogenesis signaling pathways. We speculate that NDRG1 may be involved in metabolic processes related to ferroptosis. However, more experimental studies still need to verify the mechanism of NDRG1 in ferroptosis. Overexpression of GRPEL2 affects the stability of mitochondrial proteins, and GRPEL2 may protect protein stability under mitochondrial oxidative stress (36). GRPEL2 is responsible for maintaining mitochondrial homeostasis through the nuclear factor-kappa B pathway, which affects the cell cycle in HCC (37). In particular, the composition of Fe.S-containing proteins is related to DEGs, may have an anti-tumor effect *via* mitochondrial-related pathways, such as complex I, OXPHOS, and mitophagy. Our finding suggested that upregulated NDRG1 could be related to poor prognosis through mitochondrial-related proteins in HCC. On the basis of the results of available datasets, we found that NDRG1 silencing was associated with various tumor biological functions such as RNA splicing, histone modification, and peptidyl-lysine modification. We concluded that the ferroptosis-related model was associated with the prognosis of HCC. These 3 genes may be essential targets and regulatory proteins in the biological progression of HCC. Using drug sensitivity analysis, we found a significant negative association of multiple anti-cancer drugs with the model. Multiple post-transcriptional regulatory mechanisms may be involved. Among them, Axitinib is an anti-tumor agent used clinically in adult patients with progressive renal cell carcinoma (RCC) who have failed prior treatment with a tyrosine kinase inhibitor or cytokine. This study provides some theoretical reference to determine whether Axitinib can be administered to patients with HCC based on ferroptosis-related prediction models. However clinical trials are still required to validate this theory. We also included a clinical factor, tumor size, in the prognosis model. Patients with more extensive tumor volumes have a worse prognosis, because the tumor has adapted to the body’s growth or because the tumor is discovered late. Therefore, the current research results suggest that EZH2, GRPEL2, NDRG1, and tumor size are reliable prognostic indicators.

The pan-cancer analysis results suggest that the eight hub genes (TXNRD1, TUBG1, SF3B4, PPM1G, PIGU, NDRG1, GRPEL2, and EZH2) were differentially expressed in gastrointestinal tumors. These eight genes may have unique roles in the digestive tract and are relatively stable in genomics. Cirrhosis-related HCC characteristics of HBV infection have a particular tumor immune microenvironment. This microenvironment may be in a state of immunosuppression over a long period, may play a unique role in promoting HCC to escape host immune surveillance (38). Therefore, we speculate that prognosis-related genes could affect the prognosis through the tumor immune microenvironment in HCC. However, further studies are required to determine the mechanisms of interaction between hub genes and immune cells. We also wanted to determine the role of the tumor microenvironment and energy metabolism in HCC. One of our study aim was to detect an unknown mechanism that links programmed cell death, and mitochondrial metabolism in HCC. Therefore, we performed an immune infiltration analysis and a GSEA analysis. In the immune infiltration analysis, we found that expression of the eight hub genes (TXNRD1, TUBG1, SF3B4, PPM1G, PIGU, NDRG1, GRPEL2, EZH2) and the immune infiltration of T helper cells, Tcm cells, and Th2 cells were related to HCC. Studies have shown that the infiltration of multiple immune cells plays a role in HBV-related HCC (39). T cells may mediate cell death by various mechanisms. Previous studies have demonstrated that Th17 cells may activate and induce cell death by Fas-mediated apoptosis, which is consistent with our results (40, 41).

Ferroptosis is an iron-dependent regulatory form of cell death caused by excessive lipid peroxidation, which is associated with the occurrence and response to treatment of various types of tumors. Ferroptotic injury triggers inflammation-related immunosuppression in the tumor microenvironment, which favors tumor growth. Ferroptosis can also lead to the activation of the Renin-angiotensin system, TP53 and other signaling pathways, which then leads to poor survival of patients with HCC (42). We constructed a model in which EZH2, TUBG1, and PPM1G were strongly correlated with the FANCD2. FANCD2 is an intersection protein associated with multiple ferroptosis in clear cell renal cell carcinoma and bladder cancer (43, 44). According to our results, EZH2, TUBG1, and PPM1G may play a role in ferroptosis through the key gene FANCD2. Fe.S-containing proteins were also significantly correlated with the eight hub genes. Current research has shown that Fe.S proteins participate in vital pathways such as oxidative phosphorylation and iron metabolism. Fe.S proteins are involved in the critical steps of the pathway that involve the maturation of mitochondrial proteins. The vital predisposing factors for ferroptosis are iron leakage, lipid peroxidation, and increased ROS (45, 46). The suppression of ferroptosis may contribute to tumor progression and survival (47). Some scholars believe that mitochondria play a crucial role in ferroptosis caused by erastin or cystine deprivation. However, a lack of mitochondria is affected by erastin concentrations, leading to cell death, which suggests that mitochondria may participate in ferroptosis through other currently unknown mechanisms (48). Because of that, ferroptosis mediates various tumor effects. In different damage-associated molecular patterns, ferroptosis can protect cells from drug damage (42). Such mechanisms may aggravate the damage caused by immune cells (49). Therefore, we speculate that the development of HCC may be affected by ferroptosis *via* mitochondrial metabolism and ROS. Additional mechanistic studies are still required to confirmed this possibility.

On the basis of the above-mentioned verification results of our model’s function, ferroptosis, mitochondrial metabolism, ROS, and immune infiltration are closely related to the inflammatory background of HCC. The background of hepatitis B infection and chronic inflammation exhibits a unique immune microenvironment (50, 51). Inflammation is tightly associated with cell death (52). Functional studies have shown that clinical parameters and inflammation are positively correlated with the prognostic models. Programmed cell death is affected by chronic inflammation, which results in tissue DNA damage (53). This study showed that our model was positively correlated with inflammation and DNA damage repair. Therefore, this model can reflect the inflammation associated with cell death in HCC. mTOR complex I can regulate endoplasmic reticulum (ER) stress (54). Transcription factor families, such as Myc and E2F, target various tumorigenesis and mediate inflammatory progression (55). Therefore, our model can reflect the prognosis of HCC in the context of inflammation.

There are some limitations to our study. First, this study recruited 104 patients with HCC in East China. This sample size was limited to a specific race and a single center. To apply our findings in the clinic, multicenter studies with a larger sample size are required. In particular, whether our prognostic model can be used as a biomarker in a large sample size needs to be investigated. Second, we developed a prognostic model for cell death associated with ferroptosis. However, several mechanisms have not yet been fully determined, and more evidence is required. In particular, ferroptosis-related therapeutic regimens should be designed for these targets and validated in *in vivo* and *in vitro* studies. Finally, further experiments are still required to verify the mechanism of action of FANCD2 through ferroptosis affecting the inflammatory and immune microenvironment of HCC. The most appropriate treatment required for patients with a high risk of recurrence and the most suitable types of surgery or adjuvant treatment required to prolong patients’ overall survival are still unknown.

In conclusion, we used bioinformatics analysis methods to predict DEGs for the occurrence and development of HCC and found eight hub genes (EZH2, GRPEL2, PIGU, PPM1G, SF3B4, TUBG1, TXNRD1, and NDRG1). We further verified the clinical tissue sample results to develop a reliable nomogram, which could be used as a prognostic model for HCC. These hub genes are involved in mitophagy, OXPHOS, and Fe.S-containing proteins. EZH2, TUBG1, and PPM1G are related to the ferroptosis gene, FANCD2, and may be involved in tumor biological behaviors mediated by ferroptosis. The background of chronic hepatitis may play an essential role in the development of HCC. According to our data set, three genes, EZH2, GRPEL2, and NDRG1, and one clinical factor, tumor size, could be used as reliable indicators for evaluating the prognosis in patients with HCC.

## Data availability statement

Publicly available datasets were analyzed in this study. This data can be found here: The Cancer Genome Atlas (TCGA) database.

## Ethics statement

The studies involving human participants were reviewed and approved by the Human Ethics Review Committee of The Cancer Hospital of the University of Chinese Academy of Sciences (Zhejiang Cancer Hospital). The Ethical Review Committee number of this study was IRB-2021-234. The patients/participants provided their written informed consent to participate in this study.

## Author contributions

YZ, JW, FH made substantial contributions to conception and design; FH and YZ designed the study and performed the analysis; XZ and LS acquired the data, YC and FH performed the analysis and interpretation of data; FH, YX, LX, LS drew the Figures and tables; FH and DC took part in drafting the article or revising it critically for important intellectual content; FH and YZ gave final approval of the version to be published. XC gave a direction of this study and took part in the manuscript. XC, FH, YZ gave financial support through their own research funds. All authors contributed to the article and approved the submitted version.

## Glossary

**Table:**

HCC or LIHC	Hepatocellular carcinoma
FPKM	Fragments Per Kilobase of exon model per Million mapped fragments
TCGA	The Cancer Genome Atlas
GO	Gene Ontology
KEGG	Kyoto Encyclopedia of Genes and Genomes Enrichment Analyses
LASSO	least absolute contraction and selection operation
GSVA	the Gene Set Variation Analysis
CC	cellular components
MF	molecular function
BP	biological pathways
DEGs	The Differentially Expressed Genes
HRP	horseradish peroxidase
AFP	Alpha-fetoprotein
ALT	Alanine aminotransferase
HBsAg	Hepatitis B surface antigen
ALT	Alanine aminotransferase
OXPHOS	oxidative phosphorylation
HIAC	hepatic arterial infusion chemotherapy
FLV	inadequate future liver volume
OS	overall survival
KIRP	kidney renal papillary cell carcinoma
KICH	kidney chromophobe
LGG	brain lower-grade glioma
GBM	glioblastoma multiforme
BRCA	breast cancer
LUSC	lung squamous cell carcinoma
LUAD	lung adenocarcinoma
READ	rectum adenocarcinoma
COAD	colon adenocarcinoma
UCS	uterine carcinosarcoma
UCEC	uterine corpus endometrial carcinoma
OV	ovarian serous cystadenocarcinoma
HNSC	head and neck squamous carcinoma
THCA	thyroid carcinoma
PRAD	prostate adenocarcinoma
STAD	stomach adenocarcinoma
SKCM	skin cutaneous melanoma
BLCA	bladder urothelial carcinoma
CESC	cervical squamous cell carcinoma and endocervical adenocarcinoma
ACC	adrenocortical carcinoma
PCPG	pheochromocytoma and paraganglioma
SARC	sarcoma
LAML	acute myeloid leukaemia
PAAD	pancreatic adenocarcinoma
ESCA	oesophageal carcinoma
TGCT	testicular germ cell tumours
THYM	thymoma
MESO	mesothelioma
UVM	uveal melanoma
DLBC	lymphoid neoplasm diffuse large B-cell lymphoma
CHOL	cholangiocarcinoma
