# Supplementary material for: Construction and validation of a prognostic model for hepatocellular carcinoma: Inflammatory ferroptosis and mitochondrial metabolism indicate a poor prognosis
Source: Front Oncol. 2023 Jan 5;12:972434. doi: 10.3389/fonc.2022.972434 (PMC9850107; doi:10.3389/fonc.2022.972434)
Supplement: Supplementary file 2 [file Image_1.pdf]

Supplementary Figures

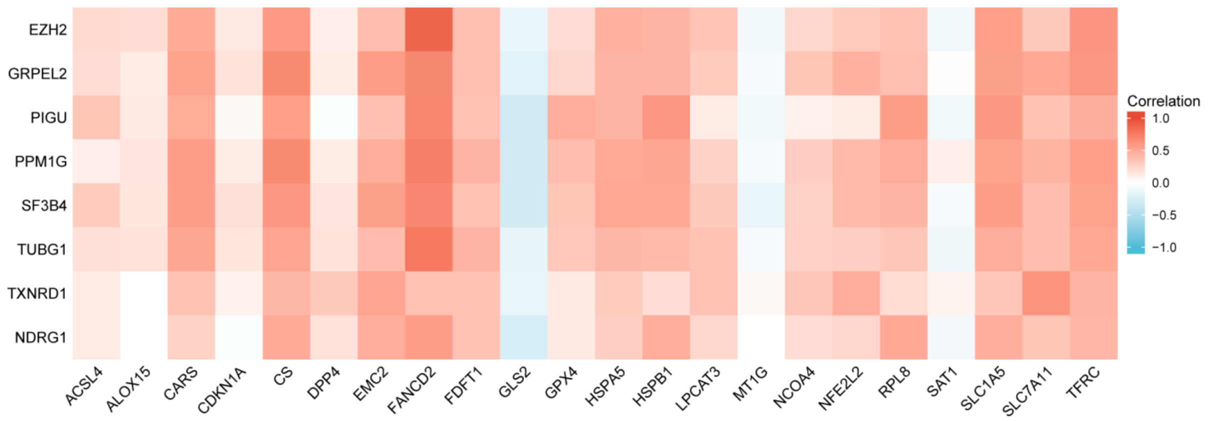

Supplementary Figure 1: Correlation between Ferroptosis related gene signature and Hub genes

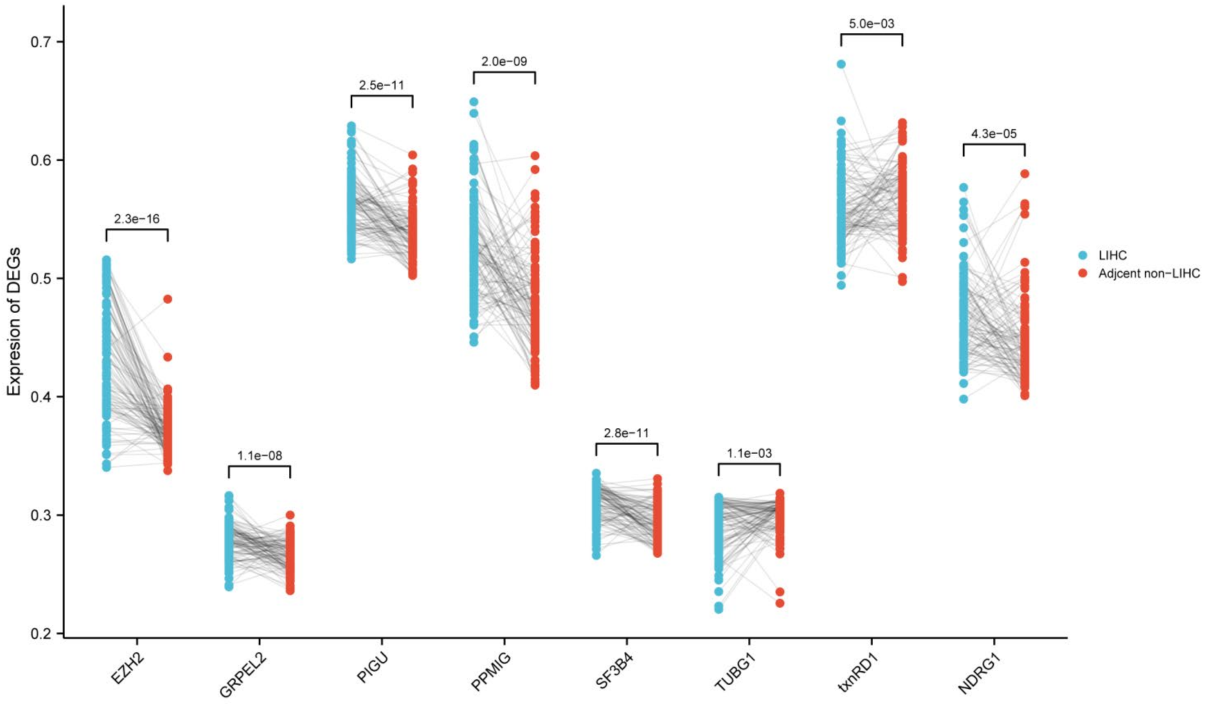

Supplementary Figure 2: Quantitative expression levels of eight hub genes by tissue chip immunohistochemistry

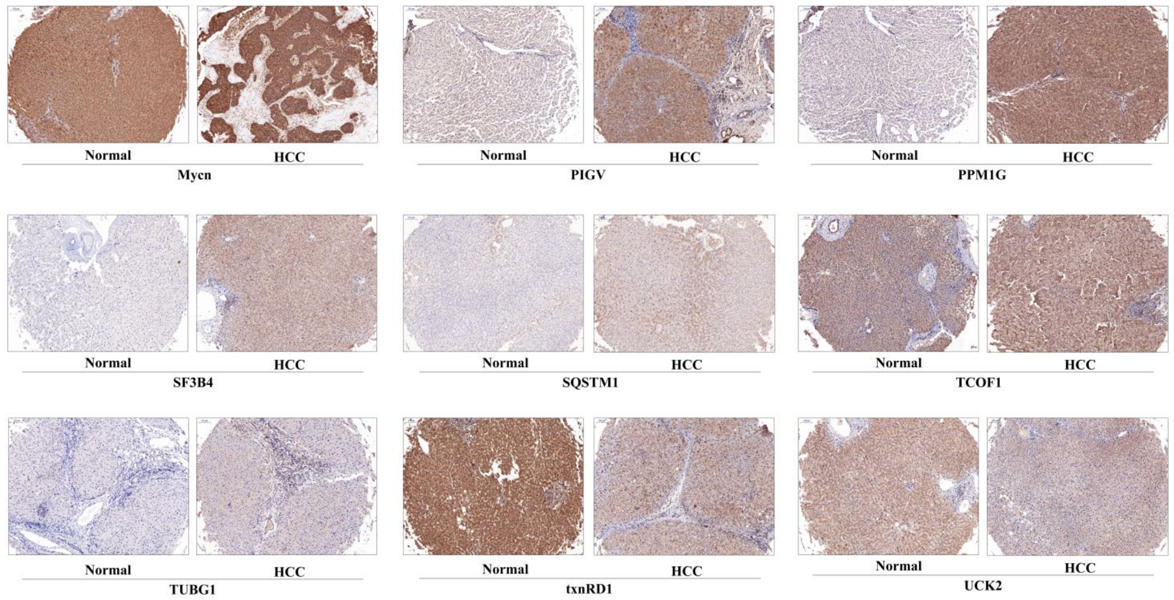

Supplementary Figure 3: Characteristic image of immunohistochemical tissue microarray staining (supplement image of Figure 5D)

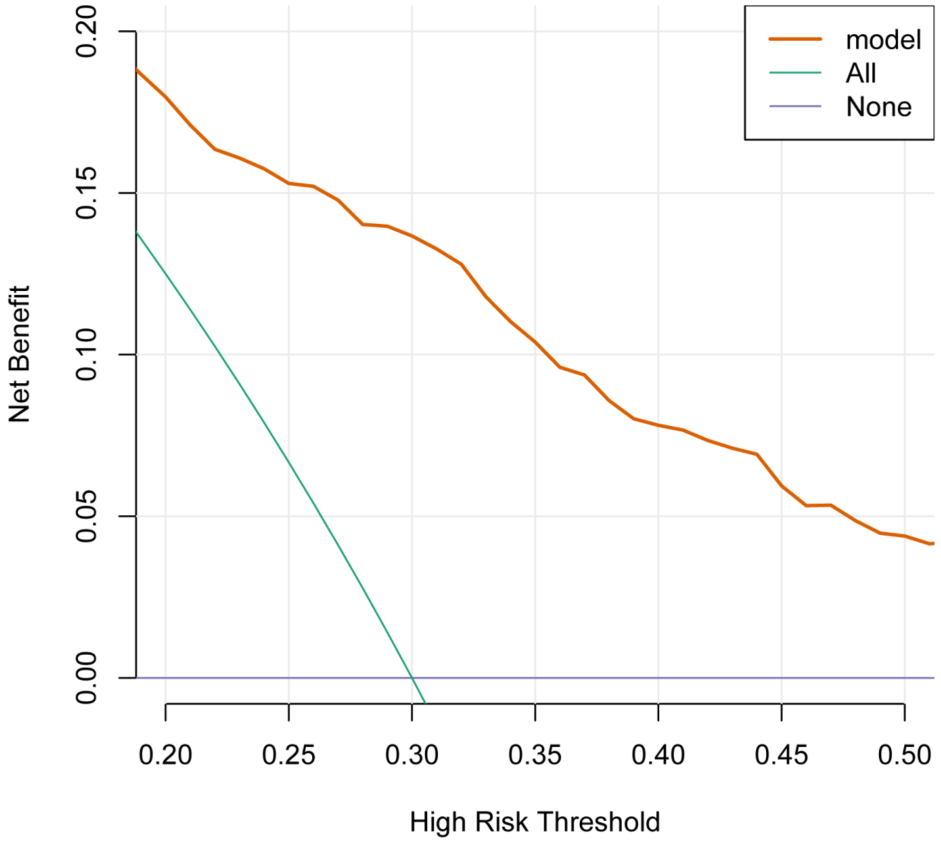

Supplementary Figure 4: C-index to evaluate our model. C-index of this model is 0.811(0.797-0.825).

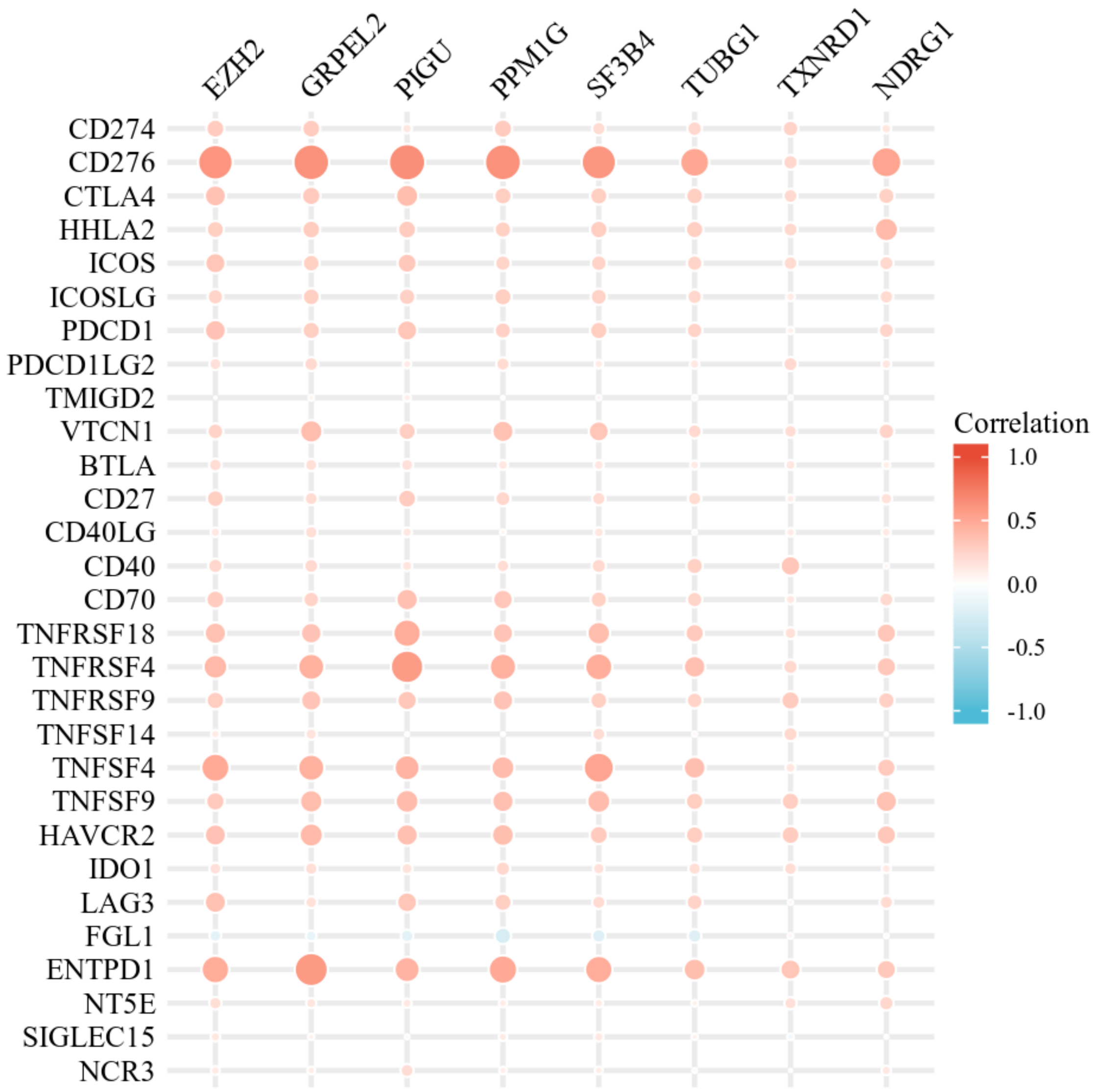

Supplementary Figure 5: Correlation analysis of the expression of 30 immune checkpoints. It include B7-CD28 family (CD274, CD276, CTLA4, HHLA2, ICOS, ICOSLG, PDCD1, PDCD1LG2, TMIGD2, VTCN1), TNF superfamily (BTLA, CD27, CD40LG, CD40, CD70, TNFRSF18, TNFRSF4, TNFRSF9, TNFSF14, TNFSF4, TNFSF9), and other immune checkpoint (HAVCR2, IDO1, LAG3, FGL1, ENTPD1, NT5E, SIGLEC15, VSIR, NCR3)
